# Penile Length can be Estimated by the Foot-Length? Study in Human Fetuses with Neural Tube Defects

**DOI:** 10.1590/S1677-5538.IBJU.2025.9922

**Published:** 2025-12-30

**Authors:** Moyses E. Mizrahi, Ricardo C. de Mattos, Carla M. Gallo, Francisco J. B. Sampaio, Luciano A. Favorito

**Affiliations:** 1 Universidade do Estado do Rio de Janeiro Unidade de Pesquisa Urogenital RJ Brasil Unidade de Pesquisa Urogenital, Universidade do Estado do Rio de Janeiro, Uerj, RJ, Brasil

**Keywords:** Penis, Neural Tube Defects, Humans

## Abstract

**Background:**

There are no reports comparing penile length with foot-length between normal and anencephalic fetuses.

**Aim:**

To compare the penile length with foot-length in fetuses with anencephaly and without anomalies.

**Materiais and methods:**

We studied 32 fetuses without anomalies, aged 11-22 weeks post-conception (WPC) and 13 anencephalic fetuses, aged 13-19 WPC. We evaluated penile free portion length and width, penile root length and width and total penile length with a digital caliper and the aid of computer programs (Image Pro and Image J). The Shapiro-Wilk test was employed to ascertain the normality of the data and to compare quantitative data between normal vs. anencephalic fetuses. Simple linear correlations were calculated for penile measurements according to foot-length.

**Outcomes:**

This is a morphometric study of human fetuses using a standardized technique to measure the penis in human fetuses.

**Results:**

Total penile length varied from 4.69 to 29.77mm (mean =15.67) in normal fetuses and from 7.49 to 18.46mm (mean=11.48) in anencephalic fetuses without significant differences. The linear regression analysis indicated that the total penile length has a strong and significant correlation with the foot length in the control group (r2=0.8505, p<0.001) and a moderate correlation of total penile length and foot length in the anencephalic group (r2=0.6813; p=0.0032) and the penile body and root width increased significantly and positively with fetal foot length in normal and anencephalic fetuses.

**Clinical Implications:**

This study may suggest a correlation between foot size and penis size in human fetuses during the 2nd gestational trimester of development.

**Strengths & Limitations:**

Sample size was small; however, anencephalic fetuses are rare, so observations of a small sample are still relevant.

**Conclusions:**

Penile length increased significantly and positively when correlated with foot length during the 2nd trimester of gestational development. We can suggest that foot size can be considered an indicator of penis size in human fetuses.

## INTRODUCTION

Penile size has been suggested to associate with sexual strength, virility, and vitality in men, as well as a man's self-esteem (1, 2). Medical consultations related to penis size are very common at pediatric, urology and endocrinology clinics, because the issue has significant medical, sexual, psychological and social relevance (3, 4). There is no indication that penis size differs between ethnicities (5).

Perceptions of penis size are culture specific. The males of ancient Greece believed that small penises were ideal. Large penises in Greek art are reserved exclusively for comically grotesque figures (6). Ancient Egyptian cultural and artistic conventions generally prevented large penises from being shown in art, as they were considered obscene (7).

Several studies measured the penile size and correlated with several body parameters like nose size, height and digit ratio (2D:4D) (8-10). Some studies analyzed the relationship between nose size and penile size (8). Height shows a weak-but-real relationship with penis length (9). A lower digit ratio (ring finger longer than index finger) has been linked to longer stretched penile length (10), but even these correlations are modest and insufficient for making accurate individual predictions.

Anencephaly is the worst form of neural tube defects and can work a model of impairment of the pelvic nerves and their development. The structure of the penis in anencephalic fetuses did not differ from that of fetuses without anomalies in previous studies (11).

There are no reports comparing penile length with foot-length between normal and anencephalic fetuses during human fetal development. Our hypothesis was that there are no differences between anencephaly and normal fetuses penile development during the human fetal period. The objective of the study was to compare the penile length and penile width with foot-length in fetuses with anencephaly and without anomalies.

## MATERIALS AND METHODS

This study was carried out in accordance with the ethical standards of the hospital's institutional committee on human experimentation. (IRB: 2.475.334, CAAE: 91095525700005259).

We studied 45 human fetuses (32 without apparent anomalies and 13 anencephalic), aged 10-22 weeks post-conception (WPC) during the period from July 2023 to October 2025, which had been aborted due to hypoxia and therefore for causes unrelated to the urinary tract. The fetuses came to our laboratory as donations from the obstetric section of our hospital. The fetuses in the control group were macroscopically well preserved, with no signs of malformation, and the stillbirth was due to hypoxia. The gestational age was determined in WPC according to the foot-length criterion. This criterion is currently considered the most acceptable parameter to estimate gestational age (12-14). The fetuses were also evaluated regarding total length (TL), crown-rump length (CRL) and body weight immediately before dissection. For the evaluation of the Total length (TL) we used a metric tape and the measurement was performed from the most prominent point of the skull to the calcaneus. The same observer performed all measurements.

Using a standardized technique, the fetuses were dissected with extraction of the pelvis "en bloc" and then identified according to gestational age and date of dissection. The pelvis blocks were then reserved in a formalized container until the moment of microdissection performed in our laboratory. The fetuses were carefully dissected with the aid of a microscope (Zeiss Discovery V8 microscope with stereoscopic lens with 16/25X magnification). The pelvis was opened to expose and identify the urogenital organs and separate the genital and urinary tracts. All fetuses were dissected under identical conditions by the same researcher, who has practical experience in microsurgery.

**Figure 1:**
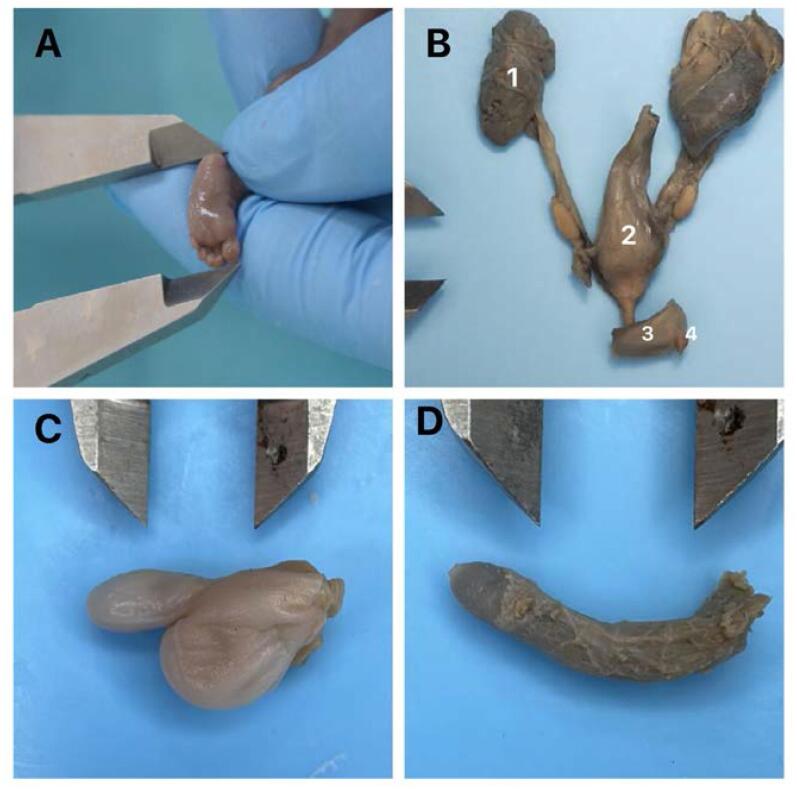
Foot length and penile measurements.

After dissection the penile total length and width, penile root and penile body length and width were measured with a digital caliper and the aid of computer programs (Image Pro and Image J) photographs were taken by the camera attached to the microscope (Zeiss Axiocam 506 Color, 6 megapixels), and the images were stored in a TIFF file (Figure-1). The biometric parameters were recorded and measurements were performed by the same observer using the Image J software, version 1.46r, because of the high intra-observer precision compared to inter-observer analysis (15, 16). The data were expressed in millimeters.

A) Measurement of foot length with a digital caliper; B) The figure shows the urogenital block after fetal dissection, 1 - Right kidney, 2 - Bladder, 3 - penile root and 4 - penle body; C) We can observe the penis of a fetus aged 16 weeks post-conception during the measurement of penile body and D) Dissection of penile root of the same fetus with 16 weeks post conception to measure penile root.

### Statistical Analysis

All parameters were statistically processed and graphically described. The Shapiro-Wilk test was employed to ascertain the normality of the data and to compare quantitative data between normal vs. anencephalic fetuses. Simple linear correlations (r^2^ values less than 0.4 reflect very weak correlation, while r^2^ between 0.4 and 0.7 reflect moderate correlation and r^2^ greater than 0.7 indicates strong correlation) were calculated for penile and fetal measurements. Statistical analysis was performed with the GraphPad Prism program (Version 6.01).

## RESULTS

Findings regarding fetal age, weight, crown-rump length, total length and penile measurements in normal and anencephalic fetuses are shown in Table-1. Mean gestational age of the normal group was 15.8 WPC, while for the anencephalic group it was 15.4 WPC, with an overall variation between 12 and 22 WPC.

**Table 1:** The table shows the analyzed parameters in normal and anencephalic human fetuses and penile measurements. WPC=weeks post-conception, SD=standard deviation.

Parameter	Normal Fetuses (13 to 19WPC)	Anencephalic Fetuses (11 to 22WPC)	p value
Weight	16 to 525 g (mean=208.91 /SD±139.3)	32 to 248g (mean=116.16 /SD±61.1)	p<0.05
Total Length	9.5 to 30 cm (mean=21.14/SD±5.71)	12 to 22cm (mean=16.96/SD±2.54)	p<0.05
Crown Rump Length	6.5 to 20.5 cm (mean=14.84 /SD±3.76)	7.5 to 14cm (mean=15.11 /SD±1.74)	p<0.05
Right Foot Length	9.9 to 40.1 mm (mean=24.99/SD±8.45)	15.17 to 35.81mm (mean=23.19 /SD±5.69)	**p<0.05**
Left Foot Length	10.41 to 40.36 mm (mean=25.41/SD±8.45)	16 to 36.28mm (mean=23.9/SD±5.96)	**p<0.05**
Total Penile length	4.69 to 29.77 mm (mean=15.87 /SD±6.53)	7.49 to 18.46mm (mean=11.48 /SD±3.4)	**p<0.05**
Penile Root Width	1.62 to 8.6 mm (mean=4.23 /SD±1.77)	2 to 5.56mm (mean=3.73 /SD±1)	p=0.337
Penile Root Length	2,22 to 16.75 mm (mean=9.02/SD±3.55)	4.14 to 12.23mm (mean=7.07/SD±2.59)	p<0.05
Penile Free Portion Length	1.37 to 18.16 mm (mean = 6.84/SD±3.66)	1.21 to 4.12mm (mean=2.79 /SD±1.37)	**p<0.05**
Penile Free Portion Width	0.85 to 7.73mm (mean = 3.66/SD+-3.66)	1.21 to 4.12mm (mean=2.79 /SD+-1.37)	p=0.097

**Figure 2:**
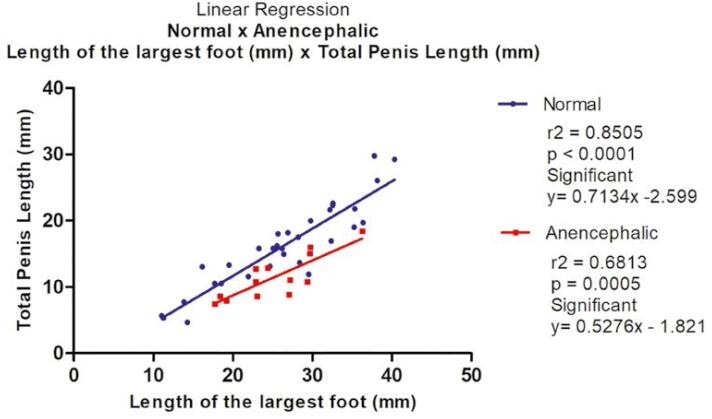
Correlation of Total penile length analyzed with fetal foot length, during the fetal period studied in normal (blue) and anencephalic fetuses (red). The points plotted represent the mean values obtained for each week studied. The linear regression analysis indicated that penile length increased significantly and positively with fetal age in normal (r2= 0.8505; p<0.0001) and anencephalic fetuses (r2=0.6813; p=0.0032).

Total penile length varied from 4.69 to 29.77mm (mean=15.67) in normal fetuses and from 7.49 to 18.46mm (mean=11.48) in anencephalic fetuses without significant differences. The linear regression analysis indicated that total penile length increased significantly and positively with fetal foot length during the 2nd gestational trimester in normal (r^2^= 0.8505; p<0.0001) and anencephalic fetuses (r^2^=0.6813; p=0.0032), a strong correlation between the total penile length and foot length in normal fetuses and a moderate correlation of total penile length and foot length in anencephalic fetuses (Figure-2).

The linear regression analysis indicated that penile body and root width increased significantly and positively with fetal foot length in normal (Penile body: r^2^=0.7076; p<0.0001; Penile root: r^2^=0.7222; p<0.0001) and anencephalic fetuses during the 2nd gestational trimester (Penile body: r^2^=0.4606; p=0.0108l; Penile root: r^2^=0.3968; p=0.0210). The r^2^ value higher than 0.7 indicates strong correlation between the penile width with foot length in normal fetuses, but the r² value below 0.4 and 0.7 reflected a moderate correlation between penile body width and foot length in anencephalic fetuses. Width of penile root in anencephalic was below 0.4 which reflected a weak correlation with foot length.

## DISCUSSION

Masculinization and penile development occur due to the influence of testosterone released by Leydig cells in response to the release of luteinizing hormone by the pituitary gland during the 1st gestational trimester (17, 18). One of the first signs of masculinization is an increase in the distance between the anus and the genital structures, followed by elongation of the penis, formation of the penile urethra from the urethral groove, and development of the foreskin (18). The human penile growth after birth occurs in two stages: the first between infancy and the age of five; and then between about one year after the onset of puberty and, at the latest, approximately 17 years of age (3, 4, 17, 18). In the present paper we studied human fetuses without anomalies and anencephalic fetuses during the 2nd gestational trimester, a very important period to estimate the formation of genital organs and the influence of neural tube defects in penile development (11, 19).

Penile size mostly linked to endocrine and genetic factors. Conditions like congenital hypogonadism or isolated gonadotropin deficiency are well documented to result in significantly smaller penises, often corrected through endocrinology treatment (20). Besides the natural variability of human penises in general, there are factors that lead to minor variations in a particular male, such as the level of arousal, time of day, ambient temperature, anxiety level, physical activity, and frequency of sexual activity (21). Compared to other primates, including large examples such as the gorilla, the human penis is thickest, both in absolute terms and relative to the rest of the body (22).

There may be a link between the malformation of the genitalia and the human limbs. The development of the penis in an embryo is controlled by some of the same Hox genes (in particular HOXA13 and HOXD13) as those that control the development of the limbs (23). Mutations of some Hox genes that control the growth of limbs cause malformed genitalia (hand-foot-genital syndrome) (24). While some minor correlations with height, nose size, or digit ratio exist, they are too weak to predict individual anatomy (3, 8, 10). Men with larger noses averaged about 5.3 in (13.5 cm) stretched vs. 4.1 in (10.4 cm) for smaller noses (8).

Stretch penile length should be interpreted in relation to anthropometric parameters in newborns, particularly body and foot length (25). Correlations between flaccid penis length, stretched out, penile circumference, height, weight, and length of the left foot were evaluated, finding low or no correlation between those mentioned, except for flaccid and stretched length (9). A previous study measured 104 men (average penile length was 3cm and the average shoe size was 9-European 43) and found no statistically significant correlation between the two parameters (21). In present paper the penile length had a significant correlation with fetal foot length during the 2nd gestational trimester.

There is currently no scientific evidence suggesting that men or boys with neurologic development disorders differ in penile size compared to neurotypical peers. Most studies on autism, ADHD, or intellectual disability focus on 2D:4D digit ratios as markers of prenatal androgen exposure, but they do not measure penile length (26) The majority of research links penile size to endocrine function and prenatal hormone exposure, not neurological development (20, 21, 24, 27).

Our study presents a comparative study about the normative parameters of penile development during the second gestational trimester in fetuses with neural tube defects. We observed some alterations in morphology of the penile development in the anencephalic group: Total penile length, penile root length and penile free portion length were significantly greater in the normal group and penile root and penile free portion width were not significantly different between the groups.

The penile length measurements were significantly greater in the normal group, demonstrating the impact of neural tube disorder on the development of the cranial-caudal axis of the penis. Previous studies show similar findings in bladder and urethra development (28). We did not find statistical significance in the penile width measurements between groups. We can speculate that the neural tube defects impair penile development during this period, but more studies (especially structural and ultrastructural studies and penile innervation) are necessary to confirm these findings.

Some limitations of our study should be mentioned: (a) the WPC of the anencephalic and control fetuses was unequal; (b) we did not conduct pathological analysis of the penis in the samples; (c) the sample size was small (however, anencephalic fetuses are rare, so observations of a small sample are still relevant); and (d) the biometric parameters of the penis were measured by a single observer, which could potentially generate measurement bias.

## CONCLUSIONS

This paper is the first to report on the correlation between penile length and fetal foot-length in human fetuses. We observe significant differences in penile length measurements in anencephalic fetuses, demonstrating the impact of neural tube defects on penile development. The penile length increased significantly and positively when correlated with foot length during the 2nd trimester of gestational development. We can suggest that foot size can be considered an indicator of penis size in human fetuses.

## Data Availability

All data generated or analysed during this study are included in this published article
